# *In situ* Synthesized Monosodium Urate Crystal Enhances Endometrium Decidualization via Sterile Inflammation During Pregnancy

**DOI:** 10.3389/fcell.2021.702590

**Published:** 2021-08-09

**Authors:** Yu-Yuan Zhu, Yao Wu, Si-Ting Chen, Jin-Wen Kang, Ji-Min Pan, Xiao-Zheng Liu, Shu-Yun Li, Gui-Jun Yan, Ai-Xia Liu, Qi-Tao Huang, Zeng-Ming Yang, Ren-Wei Su

**Affiliations:** ^1^College of Veterinary Medicine, South China Agricultural University, Guangzhou, China; ^2^Department of Obstetrics and Gynecology, Nanjing Drum Tower Hospital, Nanjing University Medical School, Nanjing, China; ^3^Department of Reproductive Endocrinology, Women’s Hospital, Zhejiang University School of Medicine, Hangzhou, China; ^4^Division of Obstetrics and Gynecology, Nanfang Hospital, Southern Medical University, Guangzhou, China; ^5^Division of Obstetrics, Foshan Maternal and Child Health Care Hospital, Southern Medical University, Foshan, China

**Keywords:** uric acid, monosodium urate crystal, decidualization, *in situ* synthesis, pregnancy

## Abstract

High level of uric acid (UA) is the major origin of gout, and is highly associated with various pregnant complications, such as preeclampsia and gestational diabetes. However, UA’s level and role in the very early stage of pregnancy has not been uncovered. This study aims to investigate the relevance of serum UA and decidualization, an essential process for the establishment and maintenance of pregnancy in women and mice during the early stage of pregnancy. In this study, we first proved that expression level of UA synthase xanthine dehydrogenase (XDH) is highly increased along with decidualization of endometrial stromal cells in both *in vitro* and *in vivo* models. Furthermore, serum and endometrial levels of UA are higher in mice with decidualized uterin horn and *in vitro* decidualized stromal cells. The existence of monosodium urate (MSU) crystal was also confirmed by immunostaining. Next, the roles of MSU on decidualization were explored by both *in vitro* and *in vivo* models. Our data shows MSU crystal but not UA enhances the decidualization response of endometrial stromal cells, via the upregulation of inflammatory genes such *Ptgs2* and *Il11*. inhibiting of Cox-2 activity abolishes MSU crystal induced higher expression of decidualization marker *Prl8a2*. At last, in women, we observed enriched expression of XDH in decidua compare to non-decidualized endometrium, the serum level of UA is significantly increased in women in very early stage of pregnancy, and drop down after elective abortion. In summary, we observed an increased serum UA level in the early stage of women’s pregnancy, and proved that the increased level of UA results from the expressed XDH in decidualizing endometrium of both human and mouse, leading to the formation of MSU crystal. MSU crystal can enhance the decidualization response via inflammatory pathways. Our study has uncovered the association between UA, MSU, and decidualization during the early stage of pregnancy.

## Introduction

Uric acid (UA) is a product of the metabolic breakdown of purine nucleotides by xanthine dehydrogenase/oxidase (XDH/XOD). Upon achieving concentrations of >0.41 mmol/L (6.8 mg/dL), UA crystalizes and forms needle-like, immunostimulatory monosodium urate (MSU) crystals, a sterile inflammatory mediator, which further cause gout, the most common form of inflammatory arthritis (Kuo et al., 2015).

In humans, risk factors for gout include diet, hyperuricemia, genetics, age, and sex (Kuo et al., 2015). The prevalence of gout and serum level of UA is lower in women than in men, but increases rapidly after menopause. However, oral administration of opposed estrogen, which combines estrogen with progestins, has been reported associated with decreased odds ratios of gout and UA level in serum of menopause women (Bruderer et al., 2015). In women at reproductive age, UA levels vary across the menstrual cycle. The lowest levels observed during the luteal phase, suggesting both estrogen and progesterone, the two ovarian steroid hormones, can promote UA excretion (Mumford et al., 2013). During pregnancy, serum UA level has been reported to fall in the first and second trimester and then increase during the third trimester till term (Johnson et al., 2011). A higher serum UA level is positively associated with disordered gestational outcomes, especially preeclampsia (Powers et al., 2006).

Decidualization is a transformation process during which the fibroblastic endometrium stromal cells differentiate into epithelial-like secretory decidual cells, which is essential for the establishment and maintenance of pregnancy (Su and Fazleabas, 2015). Failure or impairment of decidualization leads to multiple pregnancy disorders, including implantation failure, miscarriage, or preterm birth (Gellersen and Brosens, 2014). Other than ovarian steroid hormones estrogen and progesterone, many inflammatory factors and immune cells have also been reported to play essential roles during embryo implantation and decidualization (Mor et al., 2011). Recently, significant advances have been made regarding non-infectious or sterile inflammatory initiators during pregnancy (Nadeau-Vallee et al., 2016). In previous studies, we and others have shown that the endoplasmic reticulum (ER) stress and the unfolded protein response (UPR) processes are associated with decidualization and act as inducers of the sterile inflammatory response during the implantation period (Gu et al., 2016; Soczewski et al., 2020). We also reported that extracellular ATP induces decidualization of endometrial stromal cells via IL-8 in humans and mice (Gu et al., 2020a,b). However, to our knowledge, serum UA levels during the very early gestation stage have not yet been reported, and the role of MSU crystal, a well-known sterile inflammatory mediator, during the process of decidualization is still unknown.

In the present study, we observed increased UA synthetase Xdh along with decidualization in both natural pregnancy and artificial decidualization model in mice, which resulted in higher levels of UA in endometrial tissue and serum. Furthermore, our data suggested that *in situ* synthesis of UA led to the crystallization of MSU and further enhanced the decidualization process in both human cells and mouse models, through inflammatory pathways. At last, we observed higher expression of endometrial XDH expression, associated with higher levels of serum UA from women in approximately 6 weeks of gestation compared to that of non-pregnant women, suggesting the *in situ* synthesis of UA occurred in decidualized women endometrium as well.

## Materials and Methods

### Patient Sample Collection

Collection of human endometrium, decidua, and serum was carried out with the approvals of the Medical Ethics Committee of Nanfang Hospital, Southern Medical University, the Scientific Research Ethics Committee of the Drum Tower Hospital, Nanjing University Medical School, and the Ethical Committee of the Women’s Hospital, Zhejiang University School of Medicine. Serums (*n* = 15) and endometrial biopsies (*n* = 9) of the non-pregnant group were collected during a regular medical examination. Serums of the pregnant patients (*n* = 13) were collected during the first visit of antenatal care at 5.93 ± 0.71 weeks of gestation. 6 of them were sampled the 2nd time at about 2 weeks after elective abortion. Decidual tissues (*n* = 9) were collected from women undergoing an elective abortion in the first trimester of pregnancy. There are no significant differences in gravidity, parity, and BMI between the two groups. Serum levels of UA were measured by Blood Uric Acid Analyzer (Roche Applied Science, Basel, Switzerland). Tissues were fixed in 4% PFA and embedded in paraffin for further use. All the patients were written noticed before sampling. Demographic information on the patients who provided samples for this study is included in Table 1.

**TABLE 1:** Patients information.

**ID**	**Age**	**1st Sampling (Gestation Days)**	**Gravidity**	**Parity**	**BMI**	**2nd Sampling (Days After Abortion)**
P-1	27	36	2	0	25	14
P-2	22	43	1	0	21	/
P-3	20	35	1	0	23	16
P-4	26	41	2	0	20	/
P-5	22	44	1	0	22	/
P-6	23	42	2	1	23	17
P-7	24	45	3	1	22	15
P-8	25	38	1	0	24	/
P-9	28	41	2	0	20	/
P-10	31	35	3	1	20	/
P-11	20	39	2	0	23	13
P-12	23	49	2	0	21	18
P-13	25	52	1	0	23	/
NP-1	33	/	1	0	24	/
NP-2	26	/	1	0	22	/
NP-3	28	/	2	0	21	/
NP-4	32	/	1	0	20	/
NP-5	24	/	1	0	22	/
NP-6	28	/	2	1	25	/
NP-7	29	/	2	0	23	/
NP-8	30	/	1	0	23	/
NP-9	23	/	1	0	21	/
NP-10	26	/	3	1	22	/
NP-11	24	/	2	0	20	/
NP-12	25	/	2	0	21	/
NP-13	33	/	2	1	21	/
NP-14	27	/	2	0	24	/
NP-15	25	/	2	0	22	/
*p* value	0.015		0.7689	0.9999	0.958	

### Animals

All mice (ICR strain) were housed in the SPF Experiment Animal Facility with the approval of the Institutional Animal Care and Use Committee of South China Agricultural University. Mice were maintained in a temperature- and light-controlled environment (12 h light and 12 h dark cycle). Mature female mice (8–10 weeks) were mated with fertile males of the same strain to induce pregnancy; the day of the vaginal plug was marked as D1. To artificially induce decidualization, mice were ovariectomized and treated with E2 (100 ng, Sigma) for 3 days and P4 (1 mg, Sigma, United Sates) plus E2 (6.7 ng) for another 3 days, with 2 days of rest in between to mimic the hormonal environment of normal early pregnancy. 6 h after the last injection, 10 μL sesame oil (Sigma, United Sates) or 2 mg/mL MSU (Sigma, United Sates) was infused intraluminally into one or two uterine horns of mice to induce decidualization (S). The uninjected horn served as a hormonal control (Non-S). Daily injections of P4 plus E2 were continued for 4 days to maximize the decidual response, the day of injection was marked as AD1. A diagram of the artificial decidualization protocol was shown in Figure 2. For hormone treatment, mice were ovariectomized and treated with E2 (100 ng, Sigma, United Sates) for 3 days and P4 (1 mg, Sigma, United Sates) for 6 or 24 h. Mice were then sacrificed, serum UA was measured by Mouse UA ELISA Kit (Huiyan, China), and uterine tissues were collected and used for UA measurement, the rest of uterus were fixed in 4% PFA or snap-frozen for further use.

**FIGURE 1:**
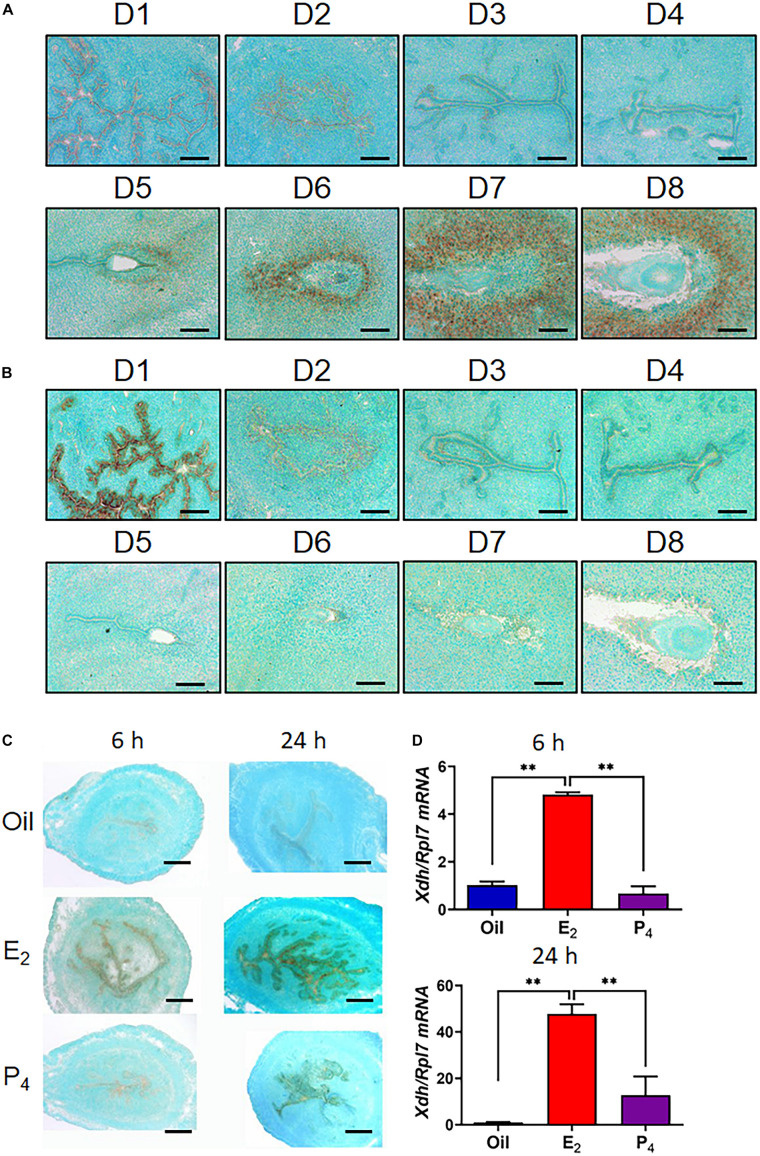
Expression of *Xdh* and *Uox* in endometrium from mouse during early pregnancy. **(A,B)**, mRNA expression of *Xdh* **(A)** and *Uox* **(B)** in the endometrium of mice on D1 to D8 of pregnancy. Expression of Xdh mRNA was significantly up-regulated by E_2_ but not P_4_ in the ovariectomized mice, detected by ISH **(C)** and qPCR **(D)**. Bar = 100 μm; ^∗^*p* < 0.05; ^∗∗^*p* < 0.01.

**FIGURE 2:**
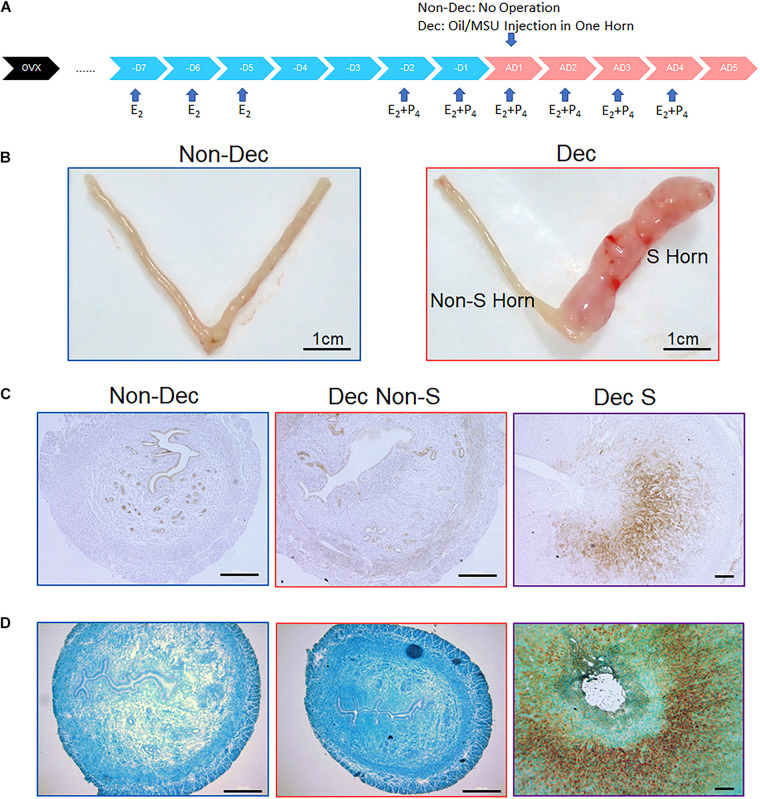
Expression of XDH protein and mRNA in the artificial decidualization mouse model. **(A)** A flow chart of protocol for inducing artificial decidualization in the mouse. **(B)** In Non-Dec mice, both horns were not stimulated by sesame oil injection. in Dec mice, one horn (S) was stimulated by sesame oil injection, and another horn (Non-S) served as the control without injection. **(C,D)** Expression of Xdh protein **(C)** and *Xdh* mRNA **(D)** in the endometrium of the non-stimulated horn (Non-S) and stimulated horn (S) of artificial decidualization mouse model, 4 days after sesame oil injection. Normal IgG and Sense-Probe were used as the negative control, respectively. Bar = 500 μm.

### Cell Culture

mESCs were isolated as follow: Uteri were washed with Hanks’ balanced salt solution (HBSS, Sigma) and then digested by digest solution [1% (w/v) trypsin (Amresco, United Sates) and 6 mg/ml dispase (Roche Applied Science, Switzerland) in 3.5 ml HBSS] for 1 h at 4°C, followed by 1 h at room temperature and 10 min at 37°C. After the luminal epithelial cells were removed by HBSS washing, the remaining tissues were digested in 6 ml of HBSS containing 0.15 mg/ml collagenase I (Invitrogen, United Sates, 17100-017) at 37°C for 35 min. The stromal cells were collected and cultured in DMEM/F12 (Sigma, United Sates, # D2906) containing 2% heat-inactivated fetal bovine serum (FBS, Biological Industries, United Sates). *In vitro* decidualization of mESCs was induced with 10 nmol/L estrogen (E2) and 1 μmol/L progesterone (P4) in DMEM/F12 containing 2% charcoal-treated FBS (cFBS, Biological Industries, United Sates) *in vitro*. HESC was obtained from ATCC and cultured in DMEM/F12 supplemented with 10% FBS, and *in vitro* decidualization of HESC was induced by treatment of the cells with 1 mmol/L medroxyprogesterone (MPA, Sigma, United Sates), and 500 mmol/L cyclic adenosine monophosphate (cAMP, Sigma, United Sates) in DMEF/F12 supplemented with 2% cFBS. Cells and culture medium were collected, and the UA levels were measured by Mouse UA ELISA Kit (Huiyan, China).

### qPCR

Total RNAs were extracted from the whole uteri or cultured cells using TRIZOL (TaKaRa, Japan) method, and cDNAs were reverse-transcribed by PrimeScript reverse transcriptase reagent kit (Vazyme, China) according to the manufacturer’s introduction. qPCR was performed using an SYBR Premix Ex Taq kit (Vazyme, China) on the CFX96 Touch^TM^ Real-Time System (BioRad, United Sates). Data were analyzed and normalized to *RPL7* for human data and *Rpl7* for mouse data. The corresponding primer sequences of each gene used in this study were listed in Table 2.

**TABLE 2:** Primer list.

**Gene**	**Accession No.**	**Primer**	**Sequence**	**Application**
*Xdh*	NM_011723.3	Probe-mXdh-F:	CGGTAGATGAGTTGGTCTTCT	ISH Probe
		Probe-mXdh-R:	CTCGATCTCCTCGACAGTAG	
*Uox*	NM_009474.5	Probe-mUox-F:	GTCCCCTGGAAACGATTTGA	ISH Probe
		Probe-mUox-R:	CTTCTATCTCAGGAAGCTGGC	
*Xdh*	NM_011723.3	qPCR-mXdh-F:	AGAGCGGACCTTGAGGGTAT	qPCR
		qPCR-mXdh-R:	GTCCTCCTCAGACTGACCCT-3	
*Prl8a2*	NM_010088	qPCR-mPrl8a2-F:	AGCCAGAAATCACTGCCACT	qPCR
		qPCR-mPrl8a2-R:	TGATCCATGCACCCATAAAA	
*Bmp2*	NM_007553.3	qPCR-mBmp2-F:	TCTTCCGGGAACAGATACAGG	qPCR
		qPCR-mBmp2-R:	TGGTGTCCAATAGTCTGGTCA	
*Wnt4*	NM_009523.2	qPCR-mWnt4-F:	TCGTCTTCGCCGTGTTCT	qPCR
		qPCR-mWnt4-R:	CTGCACCTGCCTCTGGAT	
*Rpl7*	NM_011291.5	qPCR-mRpl7-F:	GCAGATGTACCGCACTGAGATTC	qPCR
		qPCR-mRpl7-R:	ACCTTTGGGCTTACTCCATTGATA	
*Ptgs2*	NM_011198	qPCR-mPtgs2-F:	CCCCCCACAGTCAAAGACACT	qPCR
		qPCR-mPtgs2-R:	GGCACCAGACCAAAGACTTCC	
*Mpges1*	NM_022415	qPCR-mMpges1-F:	CTGCTGGTCATCAAGATGTACG	qPCR
		qPCR-mMpges1-R:	CCCAGGTAGGCCACGGTGTGT	
*Il11*	NM_001290423.1	qPCR-mIl11-F:	CTGGGACATTGGGATCTTTGC	qPCR
		qPCR-mIl11-R:	GGAGTAGCCGTTCCAGTCG	
*IGFBP1*	NM_001013029	qPCR-hIGFBP1-F:	CCAAACTGCAACAAGAATG	qPCR
		qPCR-hIGFBP1-R:	GTAGACGCACCAGCAGAG	
*RPL7*	NM_000971	qPCR-hRPL7-F:	CTGCTGTGCCAGAAACCCTT	qPCR
		qPCR-hRPL7-R:	TCTTGCCATCCTCGCCAT	

### *In situ* Hybridization (ISH)

Each probe’s cDNA template was amplified with the specific primers (listed in Table 2) and cloned into a pGEM-T plasmid (Promega, United Sates). Digoxigenin-labeled anti-sense or sense cRNA probes were transcribed *in vitro* using a digoxigenin RNA labeling kit (Roche Applied Science, Switzerland). *In situ* hybridization was performed as previously described (Hu et al., 2008). In brief, uteri were frozen sectioned into 10 mm, and then fixed in 4% (wt/vol) paraformaldehyde, permeabilized, and hybridized with each anti-sense cRNA probe (1:100) at 55°C overnight. A digoxigenin-labeled sense probe was used as the negative control. Following hybridization and post-hybridization washes, sections were incubated in an anti-digoxigenin antibody conjugated with alkaline phosphatase at 4°C overnight (Roche Applied Science, Switzerland). The signal was visualized as dark brown by incubating within a substrate solution containing 5-bromo-4-chloro-3-indolyl phosphate (Amresco, United Sates) and nitro blue tetrazolium (Amresco, United Sates). The activity of endogenous alkaline phosphatase was blocked by levamisole (2 mM, Sigma, United Sates). The sections were counterstained with 1% methyl green.

### Immunostaining

The 10 μm frozen sections were fixed in 4% paraformaldehyde for immunofluorescence and incubated in a permeabilizing solution containing 0.01% Triton X-100 (Sigma, United Sates). Sections were then blocked and incubated overnight at 4°C in an anti-precipitated UA antibody (1: 100, AB-T168, Advanced Targeting Systems, United Sates). Next, sections were incubated in respective species-specific fluorochrome-conjugated secondary antibody and then mounted with Vectashield Antifade Mounting Medium with DAPI. For immunohistochemistry, 4% paraformaldehyde-fixed, paraffin-embedded uterine tissues were sectioned into 6 μm. Following deparaffinization and hydration, sections were subjected to antigen retrieval in citrate buffer and hydrogen peroxide treatment. They were then incubated with an anti-XDH primary antibody (1: 100, Abcam, United Sates) overnight. After incubated with secondary antibody, HRP and diaminobenzidine (Vector Laboratories, United Sates) were used to visualize antigens. Sections were counterstained with hematoxylin. Normal IgG was used as a negative control to validate the specificity of antibodies.

### Western Blot

Western blot was performed as described previously (Hu et al., 2008). Briefly, proteins were extracted from cultured cells by RIPA lysis buffer, and the concentrations were calculated using the bicinchoninic acid (BCA) method (Thermo Fischer Scientific, United Sates). 10 mg protein was separated on SDS/PAGE gels and transferred onto a PVDF membrane (Millipore, United Sates). The membranes were then blocked and incubated overnight with anti-COX2 (1: 1,000, Cell Signaling Technology, United Sates) or anti-α-tubulin (1: 2,000, Cell Signaling Technology, United Sates) antibodies overnight at 4°C. After incubation with respective secondary antibodies labeled with HRP, immunocomplexes were visualized by enhanced chemiluminescence (ECL) on X-ray films.

### Alkaline Phosphatase (AP) Activity Staining

Frozen uterine sections were fixed with 4% paraformaldehyde in PBS for 10 min at 4°C and washed with 1 × PBS three times for 5 min. The uterine sections were then washed in PBS, followed by incubation at room temperature in an AP substrate solution containing 5-bromo-4-chloro- 3-indolyl phosphate (Amresco, United Sates) and nitro blue tetrazolium (Amresco, United Sates).

### Statistics

Data are expressed as means ± SEMs. Mann-Whitney test (unpaired) or Wilcoxon test (paired) was used for human data with two groups. Mouse data were analyzed using the Student’s *t*-test or one-way ANOVA, followed by Tukey’s *post hoc* multiple-range test. *p* < 0.05 was considered significant. All statistical analyses were performed by GraphPad Prism 8.0 (GraphPad Software). Power analysis was performed by using G × Power. The powers of all the human data in this study were more than 0.85.

## Results

### High Level of Xdh Expression in the Decidua of Mouse Uterus

We first explored the expression pattern of *Xdh*, the key synthetase of uric acid, in the mouse uterus from early pregnancy. There was no detectable signal in the uterus during natural pregnancy in mice, from D1 to D4 (Figure 1A). However, from D5, the time point that blastocyst implantation and decidualization occur, the signal of *Xdh* mRNA is observed to be localized in the stromal cells surrounding the implanting embryo and further diffused to the decidua zone through D6 to D8 (Figure 1A). On the other hand, mRNA expression of *Uox*, the gene that encodes urate oxidase, the metabolic enzyme of uric acid, was highly expressed on D1 of early pregnancy, but not detected in mouse endometrium from D2 through D8 (Figure 1B).

The developing embryo and the decidualized endometrial stromal cells are two major contributors to the physiological changes during early pregnancy. To test if the increase of *Xdh* expression was caused by either decidualized stromal cells or implanting embryos, we used a mouse model named artificial decidualization. In this model, mice were ovariectomized, and serial E2 and P4 were given exogenously to mimic hormone levels of early pregnancy. Then, one horn of the endometrium was then stimulated to decidualize by injection of sesame oil without the presence of an embryo (Dec S), while another horn received no stimulation and served as control (Dec Non-S) (Su et al., 2016). Meanwhile, we have experimented with an additional group of mice named Non-Dec: the hormone primed mouse without oil stimulation in either of the two horns. A diagram of this experimental design was shown in Figures 2A,B. We then examined the expression pattern of Xdh in uterine horns of Non-Dec mice and both the stimulated (S) and non-stimulated (Non-S) horns of Dec mice. The result showed higher levels of Xdh mRNA and protein in artificial decidualized endometrium (Dec S) than those in non-stimulated control horn of the same mouse (Dec Non-S) or horns from Non-Dec mice (Figures 2C,D).

Decidualization of the endometrial stromal cell was controlled by both E2 and P4 (Griffith et al., 2017). To test whether E2 or P4 induced higher expression of *Xdh*, we treated ovariectomized mice with either E2 or P4. The results showed that E2 but not P4 induced *Xdh* expression in only 6 h, indicating a regulation of the estrogen pathway on *Xdh* (Figures 1C,D).

The spatiotemporal expression pattern of *Xdh* and *Uox* mRNAs suggested that *in situ* synthesis and accumulation of uric acid very likely occurred in the endometrial stromal cells during the decidualization process.

### *In situ* Synthesis of UA in Decidualized Mouse Endometrium Led to the Crystallization of MSU

**FIGURE 3:**
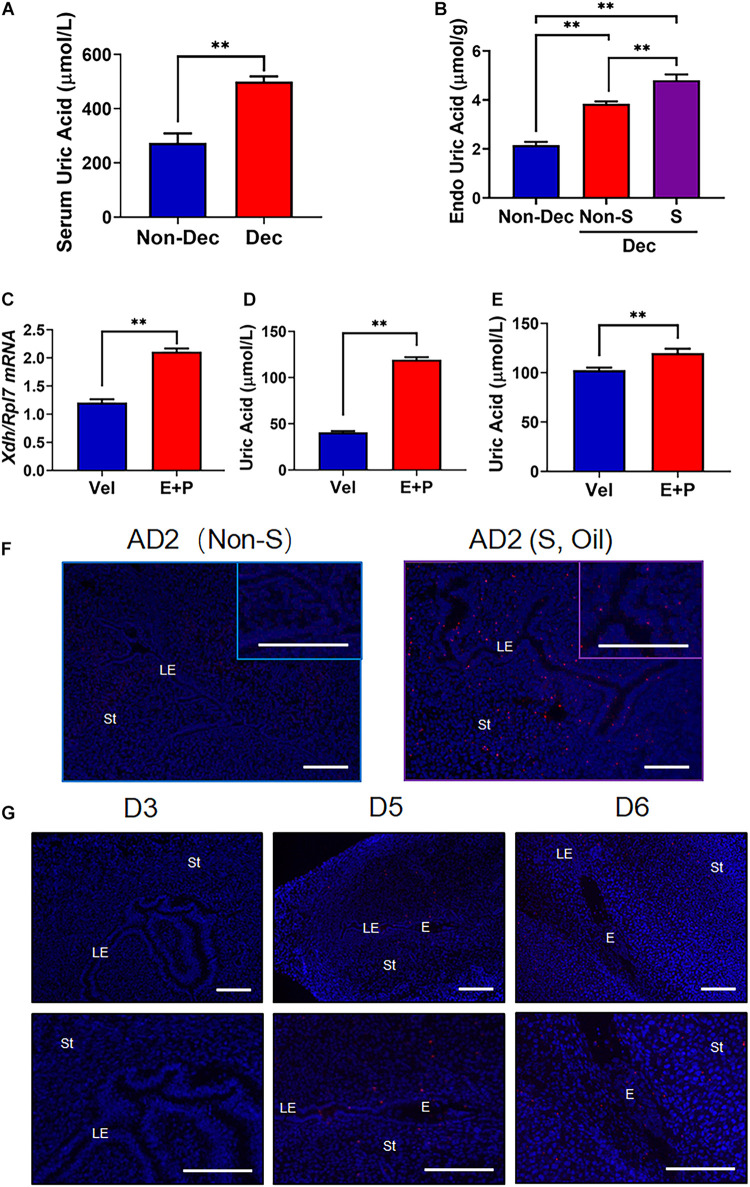
The increased level of UA and the presentation of MSU crystal in mice with decidualized endometrium. **(A)** Serum UA levels in artificial decidualized mice (Dec) and control mice (non-Dec) underwent the same hormone protocol without stimulating any of the two horns by sesame oil injection. **(B)** Endometrial tissue levels of UA in sesame oil injection stimulated decidualized horn (S) and non-stimulated horn (Non-S) of artificial decidualized mice (Dec), and Non-Dec represents uterine horns from control mice without sesame oil injection into any of the two horns. **(C)** Expression of *Xdh* in mouse endometrial stromal cells 4 days after *in vitro* decidualization (E + P). **(D,E)** UA levels in mouse endometrial stromal cells **(D)** and the supernatant of culture media **(E)** 4 days after *in vitro* decidualization (E + P). **(F)** Staining of MSU crystal in the endometrium of the non-stimulated horn (Non-S) and stimulated horn (S, stimulated by sesame oil and MSU crystal) of artificial decidualization mouse model, 1 day after sesame oil injection. **(G)** Staining of MSU crystal in the endometrium of mice on D3, D5, and D6 of pregnancy. Normal IgG was used as the negative control. Bar = 200 μm; ^∗∗^*p* < 0.01.

To test whether the higher expressed Xdh in decidua tissue could *in situ* synthesize UA and further increased the serum UA level, we measured serum UA level (sUA) and endometrial tissue UA level (eUA) in both Dec and Non-Dec groups of mice. Compared to Non-Dec mice, which received no stimulation, the sUA levels of decidualized mice were approximately twice higher (500.37 ± 18.97 vs. 273.78 ± 34.74 μmol/L, *p* < 0.01, Figure 3A). As described above, the artificial stimulation by oil injection in one uterine horn of Dec mice was the only variable between the two groups of mice. Therefore, the significant difference in serum UA levels of the two groups of mice resulted from decidualization induction, indicating the higher expression of Xdh in decidua resulted in *in situ* synthesis of UA. Furthermore, in Dec mice, endometrial tissue UA levels (eUA) were significantly higher in the stimulated uterine horn (Dec S) than the unstimulated horn (Dec Non-S) of the same mouse (4.81 ± 0.14 vs. 3.84 ± 1.09 μmol/g, *p* < 0.05, Figure 3B), further confirmed the *in situ* synthesis of UA by decidualization induced higher level of Xdh. In addition, we tested the *Xdh* expression and the levels of UA in *in vitro* decidualized mESCs. After 4 days of induction, we detected a significantly increased level of *Xdh* mRNA in the E + P treated decidualization group compares to the vehicle-treated group (Figure 3C), associated with increased UA levels in these cells (Figure 3D), as well as in the supernatant of the culture medium (Figure 3E).

A generally agreed saturation point of MSU crystallization is sUA > 410 μmol/L (6.8 mg/dL) (Dalbeth et al., 2019). To explore the possibility that the *in situ* synthesized UA results in the crystallization of MSU in decidualized endometrium, we further performed immunofluorescence using an antibody that stains explicitly precipitated but not free uric acid (van de Hoef et al., 2013). Our result showed that MSU crystal existed in the endometrium 24 h after artificial decidualization (S), but no MSU signal was detected in the endometrium without decidualization (NS) (Figure 3F). During natural pregnancy, the MSU signal is presented in the stroma surrounding the embryo, where decidualization is initiated, on D5 and D6 of pregnancy (Figure 3G). In contrast, non-decidualized endometrium from D1 through D4 showed no detectable staining of MSU antibody (D3 was shown representatively in Figure 3G). These results suggested that the high level of *in situ* synthesized UA in the decidualized endometrium led to MSU crystallization.

### MSU Crystal but Not Soluble UA Induced Decidualization of Stromal Cells *in vitro* and *in vivo*

**FIGURE 4:**
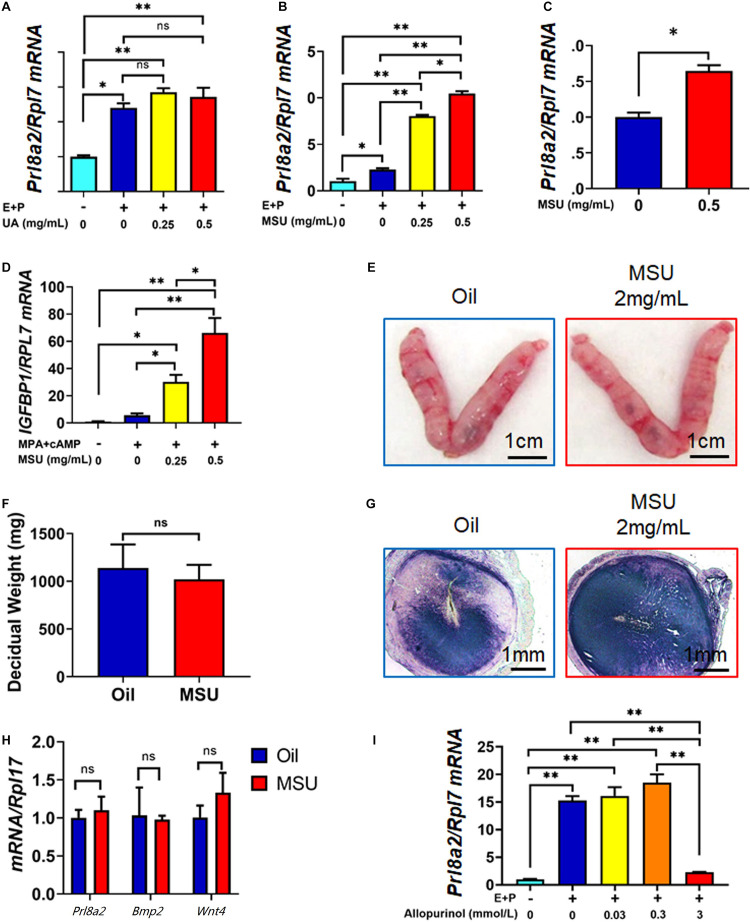
MSU but not UA enhances the decidualization response of endometrial stromal cells in humans and mice. **(A)** Expression of decidualization marker *Prl8a2* in mESC 6 h after decidualization (E + P) together with different doses of UA treatment. **(B)** Expression of decidualization marker *Prl8a2* in mESC 6 h after decidualization (E + P) together with different doses of MSU crystal treatment. **(C)** Expression of decidualization marker *Prl8a2* in mESC 6 h after 0.5 mg/mL MSU crystal treatment without decidualization. **(D)** Expression of decidualization marker *IGFBP1* in HESC 6 h after decidualization (MPA + cAMP) together with different doses of MSU crystal treatment. Morphology **(E)** and decidual weight **(F)**, AP activity staining **(G)**, and expression of decidualization markers *Prl8a2, Bmp2*, and *Wnt4* **(H)** of mouse uterus 4 days after decidualization stimulated by sesame oil (Oil) or MSU. **(I)** Expression of decidualization marker *Prl8a2* in mESC 12 h decidualization (E + P) together with different doses of Xdh inhibitor Allopurinol treatment. ^∗^*p* < 0.05; ^∗∗^*p* < 0.01; ns, *p* > 0.05.

Many inflammatory factors and immune cells have been proved to play essential roles during embryo implantation and decidualization (Mor et al., 2011). We then explored if the increased local UA level or MSU crystal, a well-known sterile inflammatory mediator, can affect the process of decidualization. To test our hypothesis, we treated mouse endometrial stromal cells (mESC) with UA or MSU crystal *in vitro*, together with a well-established decidualization cocktail (E + P). As shown in Figures 4A,B, only MSU crystal but not soluble UA was able to enhance the decidualization response of mESC in only 6 h, evidenced by the highly expressed decidualization marker gene *Prl8a2.* Furthermore, MSU crystal induced higher expression of *Prl8a2* even without combining with the E + P cocktail (Figure 4C). In addition, human endometrial stromal cells (HESC) were also used to test the effects of MSU crystal on decidualization. Similar to mESC, the response of HESC to decidualization treatment (MPA + cAMP) was significantly enhanced by MSU crystal (Figure 4D).

Next, we tested the effect of MSU crystal on decidualization *in vivo* by replacing sesame oil, the commonly used inducer in the artificial decidualization model, with MSU crystal inartificial decidualization mouse model. A total amount of 2 mg/mL of MSU crystal can perfectly mimic sesame oil in inducing artificial decidualization of hormone primed receptive endometrium. The decidual weight, alkaline phosphatase staining, and expression of decidualization marker genes *Prl8a2*, *Bmp2*, and *Wnt4* were comparable in the two groups (Figures 4E–H), indicating that MSU crystal act as an inducer for decidualization similar to sesame oil.

To further confirm that MSU is necessary for the decidualization of mESCs, we used Allopurinol, an antagonist of xanthine and hypoxanthine, to inhibit the activity of XDH, therefore which in turn reduces the level of sUA and MSU (Dalbeth et al., 2019). In our study, administration of Allopurinol could inhibit mESCs decidualization in a dose-dependent manner. When the dose reached 3 mmol/L, allopurinol treatment significantly repressed expression of *Prl8a2* in *in vitro* decidualized mESC (Figure 4I), further proved the importance of the *in situ* synthesis UA and MSU to the decidualization of mESC. These results demonstrated that higher expressed Xdh during decidualization in endometrium resulted in *in situ* synthesis UA and MSU, which in turn promoted decidualization itself.

### MSU Crystal Enhanced Decidualization Through the Up-Regulation of Inflammatory Genes

**FIGURE 5:**
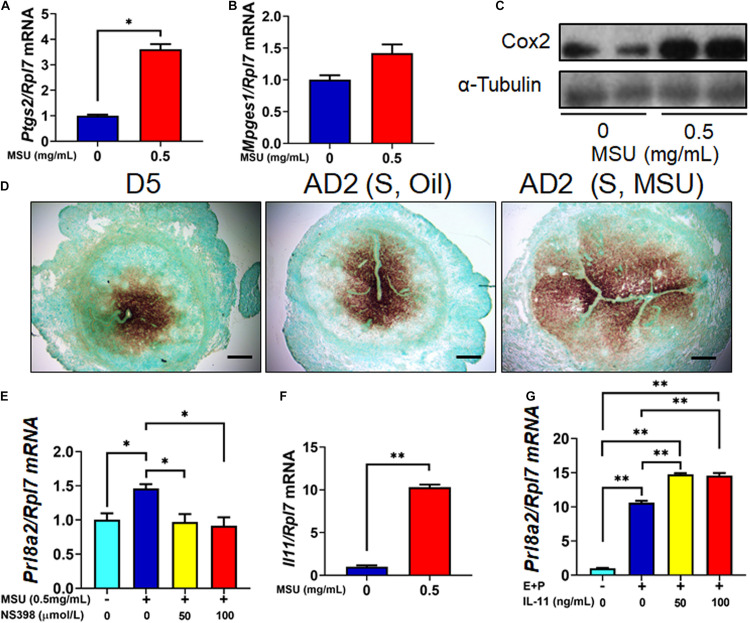
Higher expression of inflammatory genes in MSU crystal-treated mESC. **(A)** The expression level of *Ptgs2* mRNA in mESC 6 h after MSU crystal treatment. **(B)** The expression level of *Mpges1* mRNA in mESC 6 h after MSU crystal treatment. **(C)** The expression level of Cox-2 protein (encoded by *Ptgs2*) in mESC 6 h after MSU crystal treatment. **(D)** Expression pattern of *Ptgs2* mRNA in the endometrium on D5 of pregnancy, 1 day after artificial decidualization stimulated by sesame oil [AD2 (S, Oil)] or MSU [AD2 (S, Oil)]. **(E)** Expression of *Prl8a2* in after MSU crystal treatment, together with Cox-2 inhibitor NS398. **(F)** The expression level of *IL11* mRNA in mESC 6 h after MSU treatment. **(G)** Expression of decidualization marker *Prl8a2* in mESC 6 h decidualization (E + P) together with different doses of IL-11 treatment. Bar = 200 μm; ^∗^*p* < 0.05; ^∗∗^*p* < 0.01.

Many inflammatory factors have been shown as essential mediators of decidualization, such as Cyclooxygenase 2 (COX2, encoded by *Ptgs2*) and Interleukin 11 (IL-11, encoded by *Il11*) (Lim et al., 1997; Dimitriadis et al., 2002). Previously, MSU crystal was also reported to significantly stimulate Cox2 and IL-11 expression in other cell types such as osteocyte and macrophage (Scanu et al., 2015; Chhana et al., 2018; Liu et al., 2018). In this study, we tested the effect of MSU crystal in the expression of these pro-inflammatory genes. The results showed that expressions of both *Ptgs2 and Il11* were induced by MSU treatment in mESC (Figure 5A,C,F). *Mpges1*, the synthetase of Prostaglandin E, was also increased by MSU crystal treatment, but without a statistical significance (Figure 5B). Moreover, the expression pattern of *Ptgs2* in mouse decidua induced by MSU was comparable to decidua induced by sesame oil or implanting blastocyst (Figure 5D). Furthermore, NS398, an inhibitor to COX2 activity, was able to abolish the up-regulation of *Prl8a2* expression induced by MSU crystal (Figure 5E). We also confirmed the capacity of IL-11 to induce *in vitro* decidualization on mESC (Figure 5G). Our results taken together suggested that MSU may induce decidualization via these inflammatory pathways.

### Serum UA Increased Along With the Process of Decidualization in Women

**FIGURE 6:**
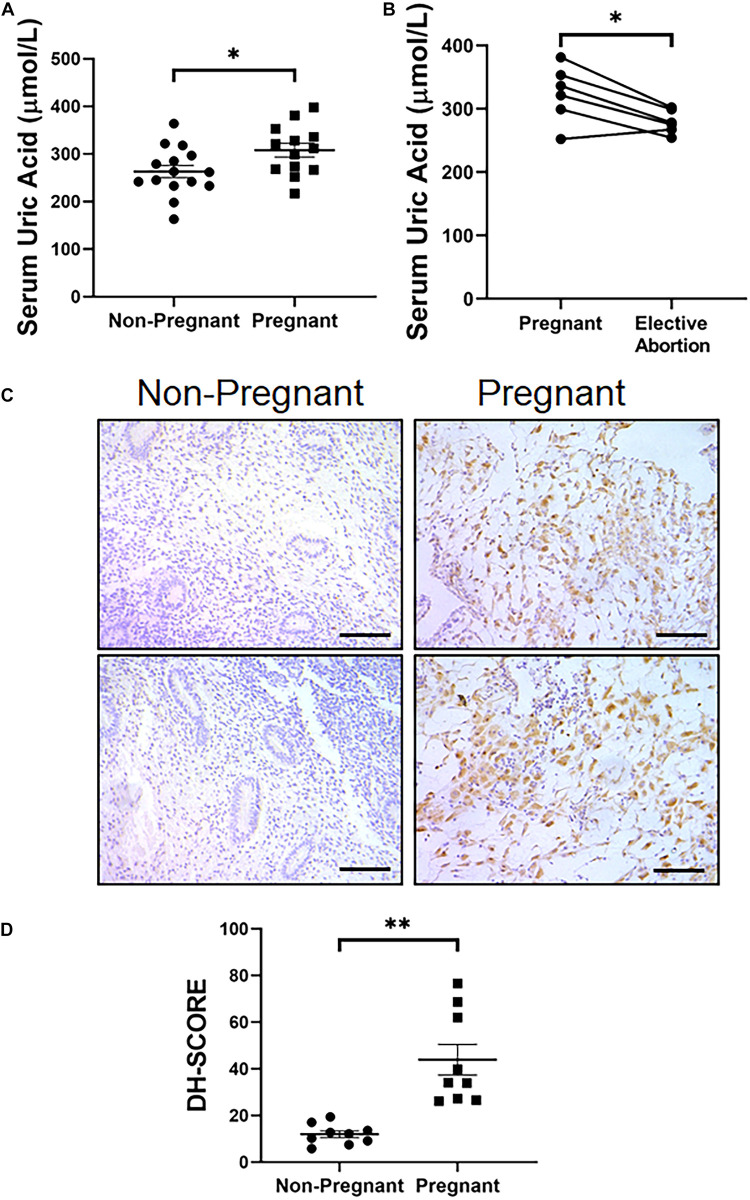
Increased serum level of UA and endometrial expression of XDH in pregnant women at gestational 5–7 weeks. **(A)** Serum level of UA in non-pregnant women and pregnant women (*n* = 15 and *n* = 13, respectively). **(B)** Serum UA levels in pregnant women at gestational 5–7 weeks and 2 weeks after elective abortion (*n* = 6). **(C)** Expression pattern of XDH protein in the endometrium of non-pregnant women and decidualized endometrium of pregnant women (Each image represents one patient). **(D)** Digital Histo-Score of XDH staining in the endometrium of non-pregnant women and decidualized endometrium of pregnant women (*n* = 9 for each group). ^∗^*p* < 0.05; ^∗∗^*p* < 0.01; Bar = 100 μm.

At last, we wondered if the *in situ* synthesis of UA in decidua was also the case in women. We first tested the expression level of XDH in decidual tissue obtained from elective abortion women, which showed dramatic higher XDH expression than non-decidualized endometrium from non-pregnant women, consistent with the observation in mice (Figures 6A,B). We then assessed serum UA (sUA) levels of women in their early stages of the first trimester of gestation (5.93 ± 0.71 weeks). As expected, the sUA levels in these pregnant women were significantly higher than those in women without pregnancy (308.15 ± 30.16 vs. 263.07 ± 29.21 μmol/L, *p* < 0.05, Figure 6C). Additionally, 6 women from the pregnant group then accepted elective abortion. About 2 weeks after the elective abortion, the sUA levels of the same set of patients went down significantly compared to the data before abortion (279.17 ± 10.69 vs. 323.67 ± 25.89 μmol/L, *p* < 0.05, Figure 6D), and were comparable to that of non-pregnant women. These data suggested a short-term increase of sUA level at the early stage of the first trimester. Taken together, our data from human biopsy suggested a similar *in situ* synthesis of UA in women decidua during early pregnancy to that in mice.

## Discussion

In women, functional estrogen and progesterone are believed to contribute to the lower serum UA level via stimulating renal clearance of UA (Bruderer et al., 2015). During the first and second trimesters of pregnancy, decreased serum UA level is considered as a result of increased hormone levels (Johnson et al., 2011). UA relevant studies in pregnancy usually consider the whole first trimester, or first 12 weeks, of pregnancy as one period, without further divide it in detail (Johnson et al., 2011). In this study, our data obtained from the early stage of the first trimester show that the serum level of UA goes up at 5–6 weeks of pregnancy, and more importantly, drops down in a subset of patients who went elective abortion. This data has enriched our understanding of the changes in the UA level during pregnancy. The difference between our study designs with the previous studies is the time points of measurement. The previous study considers the first trimester (before 13 weeks) of pregnancy as one group, and most of the samples are collected in the later stage of the first trimester (Johnson et al., 2011). However, we assess the sUA level of pregnant women in the very early stage of the first trimester, about 5–6 weeks of pregnancy, and found an increased sUA level compared to xx non-pregnancy women. Our data suggest a dramatic change in sUA level during the early and later stages of the first trimester. A detailed study may be needed in the future.

Furthermore, the mouse artificial decidualization model in this study directly proved the induction of UA level by the decidualization process. In this model, ovaries of mice were removed, and exogenous E2 and P4 were provided via subcutaneous injection to avoid the effects of different hormone levels on UA levels. All the mice in this study were caged together to make sure they were able to reach the same food and water freely. These have been done to ensure that the decidualization stimulation acted as the only variable factor between decidualized and non-decidualized mice groups. Therefore, our mouse model proved that the increased UA level in serum during the decidualization period is a direct consequence of endometrial decidualization.

In humans, the placenta is not fully formed and functional until the end of the first trimester (Knofler and Pollheimer, 2013). Before this stage, embryo implantation and decidualization are essential events in the first trimester. Decidualization refers to the differentiation process of the fibroblastic endometrial stromal cell into large, round, epithelial-like secretory cells that provide a nutritional and immune-privileged matrix essential for the implantation and development of embryos. Failure or impairment of this process results in many disorders throughout the pregnancy, including embryo implantation failure, miscarriage, and preterm birth (Gellersen and Brosens, 2014). Evident pre-decidua presents surrounding the spiral arteries around 18 days of gestation in women and further spreads to form decidua of pregnancy upon the implantation of the embryo, which occurs on 20–24 days (Su and Fazleabas, 2015). The higher level of serum UA we observed at 5–6 weeks of gestation is temporally associated with endometrium decidualization in this period. And this has been further supported by the increased expression of XDH in decidual tissue in both humans and mice. On the other hand, as a result of the high local level of UA, MSU crystal can enhance the decidualization response of endometrial stromal cells in both human and mouse cases. Together, our study suggests a positive feedback loop between MSU and decidualization.

The higher expression level of *XDH* in the endometrium has been proved due to post-ovulatory intrauterine human chorionic gonadotropin (hCG) treatment (Humaidan et al., 2012). hCG is the primary embryonic signal through which the developing embryo can dialog with the endometrium. Post-ovulatory intrauterine hCG treatment also induces decidualization in both humans and baboons, evidenced by the expression of pre-decidual marker α-SMA (Fazleabas et al., 1999; Strug et al., 2016). Therefore, the *in situ* synthesis of UA and MSU may respond to the embryonic signal.

Evolutionarily, the establishment of pregnancy in mammals, including both placental and non-placental mammals such as opossum, is considered an inflammatory response to the embryo (Griffith et al., 2017). In humans, inflammatory processes are implicated in every step of fertility, including implantation and decidualization (Nadeau-Vallee et al., 2016). In the IVF clinic, artificial induced sterile inflammation during endometrial biopsies in the spontaneous cycle can substantially increase endometrial receptivity and doubles the rate of implantation, associated with the higher expression of *XDH* (Kalma et al., 2009). Herein, we proved that, as a major inducer of sterile inflammation, MSU enhances the decidualization response of endometrial stromal cells associated with increased expression of inflammatory genes *Ptgs2* (encodes Cox-2) and *Il11*. Previously, MSU was also reported to significantly stimulated Cox2, IL-11, and other inflammation factors expression in macrophages, osteocyte cell lines, or synovial fluid cells (Scanu et al., 2015; Chhana et al., 2018; Liu et al., 2018). More importantly, these genes have been proved to play roles during decidualization by others and us (Lim et al., 1997; Dimitriadis et al., 2002). Moreover, prostaglandin E2 (PGE2), an inflammatory molecule synthesized by COX-2 and subsequent microsomal prostaglandin synthase-1 (mPges1), a well-known decidualization inducer (Pakrasi and Jain, 2008), has been shown to induce secretion of IL-11 in HESC associated with decidualization (Dimitriadis et al., 2005). On the other hand, the cytokine IL-11 is also a hemopoietic growth factor that exhibits pleiotropic biological effects, including its well-documented pro-inflammatory properties, including induction of PGE2 secretion (Morgan et al., 2004). These data suggest that the *in situ* synthesized MSU may enhance the decidualization via sterile inflammation by up-regulating a network of these inflammatory factors.

In summary, we proved the decidual expressed XDH in both humans and mice, which synthesizes UA in an *in situ* manner in decidualized endometrium, further leading to the formation of MSU crystal in the endometrium. The presence of MSU crystal then enhances the decidualization response via inflammatory pathways. Our study has uncovered the interaction between UA, MSU crystallization, and decidualization during the early stage of pregnancy.

## Data Availability Statement

The original contributions presented in the study are included in the article/supplementary material, further inquiries can be directed to the corresponding author/s.

## Ethics Statement

The studies involving human participants were reviewed and approved by the Medical Ethics Committee of Nanfang Hospital, Southern Medical University the Scientific Research Ethics Committee of the Drum Tower Hospital, Nanjing University Medical School the Ethical Committee of the Women’s Hospital, Zhejiang University School of Medicine. The patients/participants provided their written informed consent to participate in this study. The animal study was reviewed and approved by the Institutional Animal Care and Use Committee of South China Agricultural University.

## Author Contributions

Y-YZ, YW, Z-MY, and R-WS designed the study. Y-YZ, YW, S-TC, J-WK, J-MP, X-ZL, and S-YL performed the research. G-JY, A-XL, and Q-TH contributed to the new reagents/analytic tools. Y-YZ, YW, G-JY, A-XL, Q-TH, Z-MY, and R-WS analyzed the data. Y-YZ, YW, Z-MY, and R-WS wrote the manuscript. All authors contributed to the article and approved the submitted version.

## Conflict of Interest

The authors declare that the research was conducted in the absence of any commercial or financial relationships that could be construed as a potential conflict of interest.

## Publisher’s Note

All claims expressed in this article are solely those of the authors and do not necessarily represent those of their affiliated organizations, or those of the publisher, the editors and the reviewers. Any product that may be evaluated in this article, or claim that may be made by its manufacturer, is not guaranteed or endorsed by the publisher.
